# Adipose Stem Cell-Derived Apoptotic Vesicles Attenuate Hypertrophic Scarring by Targeting the CDC20/WNT Signaling Pathway

**DOI:** 10.3390/biomedicines14051083

**Published:** 2026-05-11

**Authors:** Mengyuan Jiang, Liying Cheng, Xiyuan Mao, Lu Zhang

**Affiliations:** Department of Plastic & Reconstructive Surgery, Shanghai Ninth People’s Hospital, Shanghai Jiao Tong University School of Medicine, 639 Zhizhaoju Road, Shanghai 200011, China; jmy13656307763@163.com (M.J.); drliyingcheng@126.com (L.C.)

**Keywords:** hypertrophic scar, apoptotic vesicles, adipose stem cell, CDC20, Wnt

## Abstract

**Background**: Apoptotic vesicles (ApoVs) derived from adipose stem cells (ASCs) have recently emerged as important mediators of tissue repair and are implicated in pathways relevant to hypertrophic scar (HS). Although ASCs exhibit potential in scar modulation, the therapeutic value of their apoptotic clearance products remains largely unexplored. **Methods**: In this study, we investigated the efficacy and mechanism of staurosporine (STS)-induced adipose stem cell derived apoptotic vesicles (ASCs-ApoVs) in mitigating HS. Western blot, RT-qPCR, and immunofluorescence were used to assess fibrotic markers including α-SMA, COL1A1, and COL3A1 and so on in hypertrophic scar derived fibroblasts (HS-fibroblasts). **Results**: ASCs-ApoVs significantly reduced profibrotic marker expression in HS-fibroblasts without short-term cytotoxicity. CDC20 down-regulation was identified as a critical target, through which ASCs-ApoVs suppressed Wnt/β-catenin signaling, as evidenced by the downregulation of β-catenin, c-MYC, Cyclin D1, and AXIN2. The efficacy of ASCs-ApoVs in hypertrophic scar regulation was also confirmed by the rabbit ear scar model. Furthermore, ASCs-ApoVs demonstrated notable structural and functional stability. **Conclusions**: In summary, our results established STS-induced ASCs-ApoVs as a potent multi-target strategy for hypertrophic scar regulation. Besides, the scalable production, functional stability, and favorable safety profile of ASCs-ApoVs underscore a strong promise for clinical translation.

## 1. Introduction

Local transplantation of bone marrow mesenchymal stem cells (BM-MSCs) and adipose stem cells (ASCs) has demonstrated efficacy in scar treatment [1,2]. However, several studies indicate that the majority of transplanted ASCs undergo apoptosis within days after implantation [3], suggesting that their long-term therapeutic benefits may be mediated primarily through paracrine vesicles or necrotic debris rather than by the presence of living cells.

Currently, no studies have specifically addressed the anti-scarring or anti-fibrotic effects of substances derived from necrotic or apoptotic stem cells. However, recent evidence has confirmed that dead mesenchymal stem cells (MSCs)—whether naturally formed or artificially induced—retain immunomodulatory properties comparable to those of live ones [4,5]. Based on these findings, we hypothesize that apoptotic vesicles (ApoVs) released during stem cells apoptosis may similarly regulate scarring processes, mirroring the effects observed with viable stem cells.

Interestingly, several evidence further supports our hypothesis. ApoVs from various cell types have been reported to interact with target cells, modulating diverse pathophysiological processes including inflammation [6], immune responses [7], coagulation [8], tumorigenesis [9], and tissue regeneration [10]. Specifically, ASCs derived ApoVs (ASCs-ApoVs) have been shown to promote cutaneous wound healing [11,12,13], pro-survival of ischemic flaps [14], and hair follicle regeneration [15]. Scar formation and progression are fundamentally the consequence of aberrant wound healing and abnormal tissue regeneration [16], with excessive local inflammation [17] and heightened immune responses [18]. Besides, hypertrophic scar share similarities with tumor tissue in their rich vascular supply [19], and the myofibroblasts exhibit tumor-like proliferative capacity and metabolic activity [20]. These all establish theories of scar pathogenesis align remarkably with known biological targets of ApoVs.

Given the relatively low natural secretion rate of ApoVs and the premise that a substantial quantity of vesicles is likely required to achieve a significant effect, this study artificially generated ApoVs from ASCs by staurosporine (STS) induction. We then evaluated the regulatory effects of ASCs-ApoVs on human hypertrophic scar derived fibroblasts (HS-fibroblasts) and rabbit hypertrophic scar (HS).

The Wnt/β-catenin pathway is a highly conserved regulator of cell fate and tissue homeostasis [21]. In skin scar pathogenesis, it promotes keloid cell proliferation and inhibits apoptosis via telomerase interaction [22], drives fibroblast migration and extracellular matrix (ECM) synthesis [23]. While its inhibition suppresses fibrosis in hypertrophic scar [24], making it a promising antifibrotic target. This study further investigated whether ASCs-ApoVs suppress hypertrophic scars through modulation of the Wnt/β-catenin pathway.

## 2. Methods and Materials

### 2.1. Cell Isolation and Culture

Adipose stem cells (ASCs) isolation: ASCs were isolated from liposuction tissues (*n* = 3, from Shanghai Ninth People’s Hospital, with prior informed consent obtained) by digestion with 1 mg/mL Type I collagenase (Gibco™, Thermo Fisher Scientific, Grand Island, NY, USA, 17100017) [25] in basic ASCs culture medium (OriCell™, Guangzhou, Guangdong, China, HUXMD-90011; 37 °C, 2 h, shaking). The digested tissue was then filtered through a 70-μm strainer, centrifuged (1500 rpm, 5 min), and cultured in complete ASCs medium, supplemented with 10% FBS (Gibco™, 10099141C) and 1% penicillin/streptomycin (Gibco™, 15140122). ASCs from passages 2–4 were used for experiments.

Adipose stem cells (ASCs) identification: CD34 (a hematopoietic marker, expected to be negative in ASCs; Proteintech, Wuhan, Hubei, China, 14486-1-AP) as labeled with Alexa Fluor 488 (green) and CD105 (a mesenchymal stem cell marker, expected to be positive; Invitrogen™, Thermo Fisher Scientific, Waltham, MA, USA, MA5-17041) expression was detected using Alexa Fluor 594 (red). Nuclei were counterstained with DAPI (blue; MCE, Monmouth Junction, NJ, USA, HY-D1738).

To evaluate the multilineage differentiation potential of ASCs, cells were subjected to adipogenic, osteogenic, and chondrogenic induction. For adipogenic differentiation, cells were cultured in adipogenic medium (OriCell, HUXMD-90031) for two weeks, followed by Oil Red staining to visualize lipid droplets (red). Osteogenic differentiation was induced (OriCell, HUXMD-90021) over three weeks, and mineralization was confirmed by Alizarin Red S staining (red nodules). Chondrogenic differentiation was assessed after five weeks (OriCell, HUXMD-90041) using Alcian Blue staining (glycosaminoglycans in blue) with Nuclear Fast Red counterstaining (nuclei in red) on paraffin-embedded sections of chondrogenic pellets.

Hypertrophic scar derived fibroblasts (HS-fibroblasts) isolation: Hypertrophic scar tissues were obtained from patients undergoing scar revision surgery at Shanghai ninth peoples’ hospital from 2023 to 2024, with written informed consent acquired (Number of donors: *n* = 5; Donor age range: 25–45 years old; Scar location: abdominal skin (1), neck skin (1), back skin (1), breast skin (1) and arm skin (1); Scar duration: 6–12 months post-injury). Epidermis was removed using 2 mg/mL DispaseII (Yeasen, Shanghai, China, 40104ES80; 4 °C, 12 h, shaking), followed by dermis dissociation. Dermis was then cut into small pieces and digested with 1 mg/mL Type I collagenase (37 °C, 2 h, shaking) [26]. The suspension was filtered (70 μm), centrifuged (1500 rpm, 5 min), and cultured in high-glucose DMEM (Gibco™, 11965092) supplemented with 10% FBS and 1% penicillin/streptomycin. HS-fibroblasts at passages 3–5 were used.

Mouse oral mucosa epithelium cells isolation: Mouse oral mucosa tissues were harvested from 3-day-old neonatal C57BL/6 mice housed in an SPF facility, and subsequently cultured in complete HD-DMEM, supplemented with 10% FBS and 1% penicillin/streptomycin.

Other cell lines: HaCaT cells (RRID: CVCL_0038) and BJ cells (RRID: CVCL_3653) were both obtained from the Cell Bank of the Committee on Type Culture Collection of the Chinese Academy of Sciences on 3 September 2024. The cell lines were confirmed to be free of contamination. Cells were cultured in HD-DMEM (Gibco™, 11965092) supplemented with 10% FBS (Gibco™, 10099141C) and 1% penicillin/streptomycin (Gibco™, 15140122), maintained at 37 °C in a humidified 5% CO_2_ incubator. Cells within 3 passages of the purchased were used for experiments.

All cells were routinely passaged at 80–90% confluence using 0.25% trypsin-EDTA (Gibco™, 25200072; 37 °C, 3–5 min).

### 2.2. ASCs-ApoVs Collection and Identification

According to our previous study [12,27], ASCs apoptosis was induced with 5 μM staurosporine (STS; MCE, HY-15141; dissolved in DMSO and diluted in serum-free DMEM medium) for 12 h. With confirmation of apoptotic morphology under light microscopy, cells were trypsinized (37 °C, 2 min) and centrifuged at 300 *g* for 10 min to remove intact cells and debris. The supernatant was further centrifuged at 3000 *g* for 10–20 min to collect ApoVs, which were then washed twice with PBS to remove residual solvable STS and other soluble components.

The protein concentration of cleaning ApoVs was quantified by BCA assay (Beyotime, Shanghai, China, P0012), adjusted to 1 mg/mL in PBS, and stored at −80 °C for long-term preservation. During cell culture, ASCs-ApoVs were added to the culture medium.

Fresh ApoVs was characterized by nanoparticle tracking analysis (NTA) and transmission electron microscopy (TEM) to confirm their size and morphology.

### 2.3. Assessment of ApoVs Uptake and Subcellular Localization

To assess cellular uptake, DiO (MCE, HY-D0969; λex/λem = 484/501 nm) labeled ASCs-ApoVs were co-cultured with HS-fibroblasts for 2 h at 37 °C. Nuclei of HS-fibroblasts were counterstained with Hoechst 33,342 (MCE, HY-15559; λex/λe = 346/460 nm) for 15 min at room temperature (RT). Confocal microscopy confirmed cytoplasmic internalization of ASCs-ApoVs, with bright-field imaging delineating cell morphology.

ASCs-ApoVs labeled with DiO: ASCs were seeded on culture dishes and grown to 80–90% confluence. After gentle washing with PBS (2–3 times), the cells were incubated with 5–10 µM DiO staining solution (MCE, HY-D0969) diluted in ASCs culture medium for 20 min at 37 °C. The staining medium was then removed, and the cells were washed three times with PBS. Fresh pre-warmed growth medium was added, and the cells were incubated for 10 min, followed by three additional PBS washes to remove unincorporated dye. DiO-labeled ASCs were then subjected to apoptosis induction (5 µM STS, 12 h) as described previously to generate DiO-labeled ASCs-ApoVs.

For subcellular localization studies, HS-fibroblasts were pre-labeled with CM-Dil (Invitrogen™, C7000; λex/λem = 553/570 nm) to visualize membrane compartments, then incubated with DiO-labeled ASCs-ApoVs for 2 h at 37 °C. Nuclei were then stained with Hoechst 33,342 (15 min, RT).

HS-fibroblasts labeled with CM-Dil: HS-fibroblasts were seeded on confocal dishes and cultured to 50–60% confluence. The cells were gently washed with PBS 2–3 times. A staining medium was prepared by adding 5 µL of CM-Dil labeling solution (Invitrogen™, C7000) to 1 mL of normal growth medium. An appropriate volume of staining medium was added to fully cover the cells, followed by incubation at 37 °C for 20 min. The staining medium was then removed, and the cells were washed three times with PBS. Subsequently, fresh pre-warmed growth medium was added, and the cells were incubated at 37 °C for another 10 min. After removing the medium, the wash step was repeated three times to thoroughly remove unbound dye. Labeled HS-fibroblasts were used immediately for subsequent ASCs-ApoVs co-culture and uptake assays.

### 2.4. Real-Time Quantitative Reverse Transcription PCR (RT-qPCR)

SYBR Green-based real-time PCR was conducted to quantify gene expression levels. Total RNA was isolated from cells using TRIzol™ reagent (Invitrogen™, 15596018CN). RNA concentration and purity were determined by Nanodrop spectrophotometer. cDNA was synthesized from 1 μg RNA using reverse transcriptase (TaKaRa, RR036A). Reactions were performed in triplicate using Probe-based master mix (TaKaRa, RR820A) on a real-time PCR system. Detailed primer information is provided in Table 1: Primer information. *GAPDH* was used as the internal reference for data normalization to ensure reliable quantification. Relative gene expression was calculated using the 2^−ΔΔCt^ method.

### 2.5. Western Blot

Cells were lysed in RIPA buffer (Epizyme, Shanghai, China, PC101) with protease inhibitors (Thermo Scientific™, Waltham, MA, USA, 78430; 4 °C, 30 min) and phosphatase inhibitors (Epizyme, GRF102; if necessary). After denaturation (100 °C, 10 min) with loading buffer (Epizyme, LT101), samples (20 μg/lane) were loaded onto the SDS-PAGE gel (FuturePAGE^TM^, Changzhou, Jiangsu, China, ET15420LGel; 4–20% 15 wells) and transferred to PVDF membranes (Amersham^TM^, Cytiva, Amersham, UK, GE10600023) using a wet transfer system. Membranes were blocked with 5% non-fat milk (Epizyme, PS112), probed with primary antibodies (4 °C, overnight) and HRP-conjugated secondary antibodies (RT, 1 h), then detected with ECL (Epizyme, Omni-ECL™ SQ201).

Protein bands were quantified using ImageJ (Fiji) software (Version 1.54g, National Institutes of Health, Bethesda, MD, USA). After background subtraction, the integrated density of each target band was measured and normalized to that of the loading control (GAPDH or β-actin). When applicable, the relative expression levels were further normalized to the control group, and the data were presented as bar graphs showing the mean ± SD from at least three independent experiments. Detailed antibody information was provided in Table 2: Antibody information.

### 2.6. Immunofluorescence

Cells on coverslips were fixed with 4% PFA (Beyotime, P0099-100 mL; 30 min, RT), permeabilized with 0.1% Triton X-100 (Beyotime, ST1723-100 mL; 10 min, RT) [28], and blocked with 5% BSA (Solarbio, Beijing, China, SW3015; 30 min, RT). Primary antibody incubation was performed overnight at 4 °C, followed by fluorescent secondary antibodies (1 h, RT). Nuclei were stained with DAPI (MCE, HY-D1738), and samples were mounted for imaging using fluorescence microscopy. Detailed antibody information was provided in Table 2: Antibody information.

For each image, Regions of Interest (ROIs) were randomly selected or manually delineated around individual cell boundaries guided by the DAPI or phase-contrast signal to ensure precise quantification. The mean fluorescence intensity for each ROI was determined using ImageJ software. Data were collected from at least 30 cells per experimental group over the course of three biological replicates.

### 2.7. Cellular Cytoskeleton Staining

The cells cultured on glass coverslips were first washed with PBS, followed by fixation with 4% PFA (30 min, RT). After fixation, the cells were permeabilized with 0.1% Triton X-100 for 10 min and stained with 1:100 FITC-Phalloidin (Solarbio, CA1620; RT, 30 min). After PBS wash, nuclei were counterstained with DAPI. Samples were mounted for imaging using confocal microscopy.

### 2.8. Scratch Wound Healing Assay

Cells were seeded in 6-well plates (Corning, NY, USA, 3516) and cultured to 100% confluency at 37 °C with 5% CO_2_. A uniform scratch was then generated along the short axis (perpendicular to the reference lines) using a sterile 200 μL pipette tip. After removing detached cells by gentle PBS washing (2–3 times), 2 mL of serum-free HD-DMEM (containing 1% penicillin-streptomycin) was added to each well. Baseline images (0 h) were captured at the predefined reference points, and cell migration was monitored at 24 h and 48 h.

The relative healing rate was quantified as: 1 − (remaining scratch area at a given time point/scratch area at 0 h) × 100%.

### 2.9. CCK-8 Assay

Cytotoxicity assessment: Cells were seeded in 96-well plates (5 × 10^3^ cells/well in 200 µL complete medium) and cultured until reaching 100% confluence. After replacing with fresh medium containing 1.0 μg/mL ASCs-ApoVs (treatment group), cells were incubated for another 24 h. The medium was then removed and cells were washed three times with PBS to eliminate potential optical density (OD) interference from ApoVs. 100 µL fresh complete medium and 10 µL CCK-8 solution (Beyotime, C0037) were added to each well, followed by 2-h incubation at 37 °C. Absorbance was measured at 450 nm using a microplate reader, with culture medium alone serving as blank control.

Relative cell viability was calculated as: (OD_treatment_ − OD_blank_)/(OD_control_ − OD_blank_) × 100%.

Cell proliferation ability test: Cells (1 × 10^3^/well) were seeded in 96-well plates. After a 24-h attachment period, a baseline measurement was taken to assess initial differences in cell numbers. Then the medium of control group was replaced with fresh medium and the treatment group was replaced with fresh medium containing 1.0 μg/mL ASCs-ApoVs. At the indicated time points (24 h, 48 h and 72 h), culture medium was refreshed with cells washed with PBS. 100 µL fresh complete medium and 10 µL CCK-8 solution was added per well. Following a 2-h incubation at 37 °C, the absorbance at 450 nm was recorded.

Raw OD values were normalized to the blank control (culture medium without cells) for each experimental group to account for inter-assay variation, using the formula: (OD_control or treatment_ − OD_blank_)/OD_blank_.

### 2.10. Stability Assessment of ASCs-ApoVs Under Simulated Clinical Conditions

To evaluate the stability of ASCs-ApoVs under repeated clinical use, ASCs-ApoVs suspended in PBS were subjected to three freeze-thaw cycles (−80 °C for 20 min/37 °C for 10 min). The temperature-cycled ASCs-ApoVs were then aliquoted into four treatment groups: ① PBS control, ② 50 μg/mL proteinase K (MCE, HY-10871), ③ 50 μg/mL RNase A (MCE, HY-129046A), and ④ 8 μg/mL DNase I (Beyotime, D7073; 37 °C, 60 min incubation). Enzyme concentrations were optimized based on established vesicle studies to ensure effective digestion while preventing false negatives [29]. Proteinase K-treated samples were analyzed exclusively by RT-qPCR. RNase A-treated samples were primarily assessed by Western blot, with RT-qPCR results as reference.

### 2.11. Flow Cytometry for Cell-Cycle Detection

Cells were harvested by trypsinization (37 °C, 2–3 min), fixed in 70% pre-cold ethanol at 4 °C overnight [30], washed twice with ice-cold PBS, then treated with RNase A (100 μg/mL) and stained with propidium iodide (PI, 50 μg/mL, 488 nm excitation/575 nm emission; Beyotime, C1052) for 30 min at 37 °C in the dark. Cell cycle distribution was analyzed using a flow cytometer (BD Lyric). Data were processed with ModFit LT software (Version 6.0; Verity Software House, Topsham, ME, USA) to quantify G0/G1, S, and G2/M phase populations.

### 2.12. RNA Sequencing (RNA-Seq) and Bioinformatic Analysis

Total RNA was extracted from cell samples using TRIzol reagent (Invitrogen) according to the manufacturer’s instructions. RNA concentration and purity were assessed using a NanoDrop 2000 spectrophotometer (Thermo Scientific), and RNA integrity was confirmed with an Agilent 2100 Bioanalyzer (version B.02.12, Agilent Technologies, Santa Clara, CA, USA). Sequencing libraries were constructed from qualified RNA samples using the VAHTS Universal V6 RNA-seq Library Prep Kit (Vazyme, Nanjing, China, NRM604). All transcriptome sequencing and subsequent bioinformatic analyses were conducted by OE Biotech Co., Ltd. (Shanghai, China).

The constructed libraries were sequenced on an Illumina NovaSeq 6000 platform to generate 150 bp paired-end reads. Raw sequencing data in FASTQ format were quality-controlled and adapter-trimmed using fastp (v0.23.4) to obtain high-quality clean reads. These clean reads were then aligned to the GRCh38 using HISAT2 (v2.2.1). The expression level of each gene was quantified as FPKM, and raw read counts were generated using HTSeq-count. Principal component analysis (PCA) was performed with R (v3.2.0) to assess the reproducibility among biological replicates.

Differential expression analysis was performed using DESeq2, with genes meeting the criteria of a false discovery rate (Q value) < 0.05 and an absolute log2 fold change (|log2FC|) > 1 defined as significantly differentially expressed genes (DEGs).

To visualize expression patterns, hierarchical clustering of DEGs was carried out using R (v3.2.0). The expression profiles of the top 30 upregulated and downregulated DEGs were displayed on a radar chart generated with the R package ggradar. Functional enrichment analysis of DEGs was performed based on the hypergeometric distribution for Gene Ontology (GO) terms and pathways from the KEGG, Reactome, and WikiPathways databases. Significantly enriched terms were identified and visualized using bar plots, chord diagrams, and bubble plots in R (v3.2.0). Additionally, Gene Set Enrichment Analysis (GSEA) was implemented using GSEA software (Version 4.3.2; Broad Institute of MIT and Harvard, Cambridge, MA, USA) by ranking all genes based on their differential expression levels and testing for the significant enrichment of predefined gene sets at the extremes of the ranked list.

### 2.13. Rabbit Ear Hypertrophic Scar Model Establishment

Healthy New Zealand white rabbits (2.5–3.0 kg, equal sexes) were acclimatized for one week. After depilating the ventral ear surfaces, general anesthesia was induced via intraperitoneal pentobarbital sodium (3%, 1 mL/kg). Under aseptic conditions, a full-thickness skin and perichondrium specimen (1.0 cm × 1.0 cm) was excised from the mid-ventral region of each ear, preserving the underlying cartilage. Wounds were left open to heal under SPF conditions. Hypertrophic scar formation was assessed 30 days post-operation based on: gross observation (elevated, firm, pink scar tissue) and H&E staining (dermal fibroblast proliferation, and disorganized collagen).

Following the establishment of hypertrophic scars, the scars were randomly divided into two treatment groups (*n* = 6 scars per group): PBS injection (30–40 μL per scar as control), and ASCs-ApoVs injection (30–40 μL per scar, 1.0 μg/mL). Starting on postoperative 30 days, the scars in each group received intralesional injections of the corresponding agent twice weekly. All injections were administered in equal volumes.

Scar stiffness was assessed in vivo by three independent blinded observers using a 0–5 scale (5 = most severe stiffness). The average of the three scores was used for each scar analysis. Scar thickness (μm) was measured from H&E-stained sections using ImageJ software, defined as the perpendicular distance from the epidermal-dermal junction to the deepest fibrotic layer. Three random sections per scar were measured, and the average value was used for each scar analysis. Fibroblast proliferation was evaluated on a 0–5 scale (5 = highest density) based on both H&E and α-SMA staining. Quantifications were performed in a blinded manner by three independent observers, and the scores for each scar were averaged to obtain a single value for statistical analysis (presented as bar charts).

### 2.14. Statistics Analysis

Continuous data are presented as the mean ± standard deviation. Prior to conducting parametric tests, the following assumptions were verified: independence between sample groups, normality of data distribution (assessed using the Kolmogorov-Smirnov test), and homogeneity of variances (evaluated by the F-test). For comparisons between two groups, an independent samples *t*-test was employed. For comparisons across three or more groups, a one-way analysis of variance (ANOVA) was applied, followed by Tukey’s Honestly Significant Difference (Tukey’s HSD) test for post-hoc pairwise comparisons. In cases where only the assumption of homogeneity of variances was violated for a two-group comparison, the Satterthwaite *t*-test (Welch’s correction) was used. If other assumptions were violated, non-parametric alternatives were adopted.

To enable cross-batch comparisons of independent experiments (including RT-qPCR and select Western blot analyses), quantitative data were normalized to their corresponding internal controls. This normalization was performed by dividing individual measurements in the treatment groups by their respective control groups, thereby defining the control as a baseline of 1. In the graphical representations, the control group is depicted as a baseline of 1 without error bars, while the normalized results in treatment groups are expressed with new mean ± standard deviation.

For all tests, a two-tailed *p*-value of less than 0.05 was considered statistically significant, denoted as follows: ns *p* > 0.05, * *p* < 0.05, ** *p* < 0.01, *** *p* < 0.001, and **** *p* < 0.0001. All statistical analyses were conducted using SAS software, version 9.4.

## 3. Results

### 3.1. The Hypertrophic Scar (HS) Regulating Properties of Adipose Stem Cell Derived Apoptotic Vesicles (ASCs-ApoVs)

To start with, we isolated and identified the adipose stem cells (ASCs) (Figure S1A,B) and the STS induced apoptotic vesicles (ApoVs). The ApoVs engulfment capability of hypertrophic scar derived fibroblasts (HS-fibroblasts) was confirmed with DiO-staining (Figure S1C,D).

**Figure 1:**
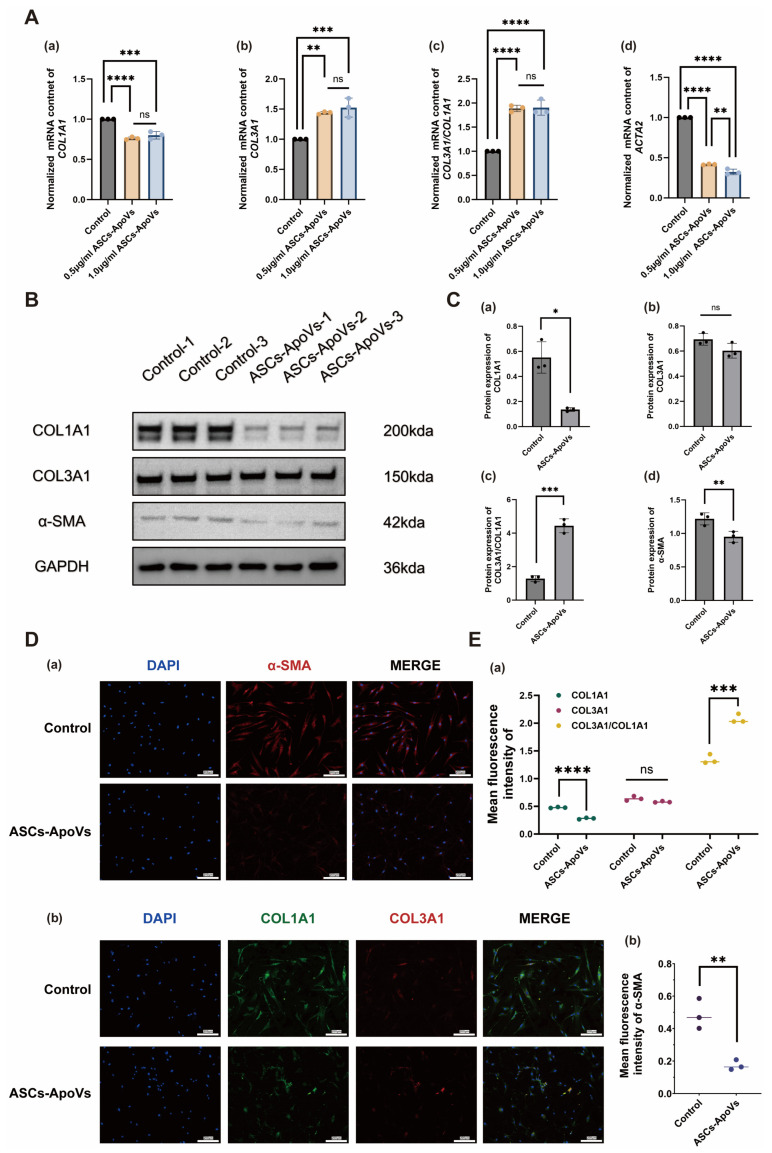
The HS-fibroblasts regulation properties of adipose stem cells derived apoptotic vesicles (ASCs-ApoVs). (**A**) Quantitative analysis of RT-qPCR results showing mRNA content of (**a**) *COL1A1*, (**b**) *COL3A1*, (**c**) *COL3A1/COL1A1*, and (**d**) *ACTA2* in HS-fibroblasts after ASCs-ApoVs treatment (1.0 μg/mL, 24 h), normalized to *GAPDH* and the control group. (Mean ± SD, *n* = 3 independent experiments, data analyzed by one-way ANOVA with Tukey’s HSD.) (**B**) Western blot analysis of myofibroblast-related markers in HS-fibroblasts after ASCs-ApoVs treatment (1.0 μg/mL, 24 h). (**C**) Quantification of (**a**) COL1A1, (**b**) COL3A1, (**c**) COL3A1/COL1A1, and (**d**) α-SMA expression from (**B**) section by relative gray vale to GAPDH. (Mean ± SD, *n* = 3 independent experiments, data analyzed by Independent-samples *t*-test.) (**D**) Immunofluorescence staining of (**a**) α-SMA (red) and (**b**) COL1A1 (green) and COL3A1 (red) in HS-fibroblasts with or without ASCs-ApoVs treatment (1.0 μg/mL, 24 h) (Scale bar = 200 μm). (**E**) Scatter plots with statistical analysis of the mean fluorescence intensity of (**a**) COL1A1, COL3A1, and COL3A1/COL1A1, and (**b**) α-SMA, normalized to DAPI intensity. (Mean ± SD, *n* = 3 independent experiments, data analyzed by Independent-samples *t*-test.) Statistical significance: ns *p* > 0.05, * *p* < 0.05, ** *p* < 0.01, *** *p* < 0.001, **** *p* < 0.0001.

Treatment with 0.5–1.0 μg/mL ASCs-ApoVs for 24 h markedly downregulated fibrotic genes, including *COL1A1* (*p* < 0.001) and *ACTA2* (*p* < 0.0001), while increasing the *COL3A1* (*p* < 0.01) transcription and *COL3A1/COL1A1* value (*p* < 0.0001) (Figure 1A). Notably, the anti-fibrotic effect on *ACTA2* was dose-dependent at the transcriptional level, with higher ASCs-ApoVs concentrations demonstrating enhanced efficacy (*p* < 0.01). Western blot with 1.0 μg/mL ASCs-ApoVs confirmed reduced COL1A1 (*p* < 0.05) and α-SMA (*p* < 0.01) expression, stable COL3A1 level, and a higher COL3A1/COL1A1 value (*p* < 0.001) (Figure 1B,C). Immunofluorescence also validated ASCs-ApoVs’ therapeutic potential in protein level (Figure 1D,E).

In summary, STS-induced ASCs-ApoVs could effectively modulate HS-fibroblasts phenotypes, exhibiting potent anti-fibrotic effects.

### 3.2. Cytotoxicity Evaluation of ASCs-ApoVs at Biologically Active Concentration

To determine whether ASCs-ApoVs’ scar modulating effects at therapeutic concentrations involve cytotoxicity and mobility regulation, we conducted complementary functional assay.

**Figure 2:**
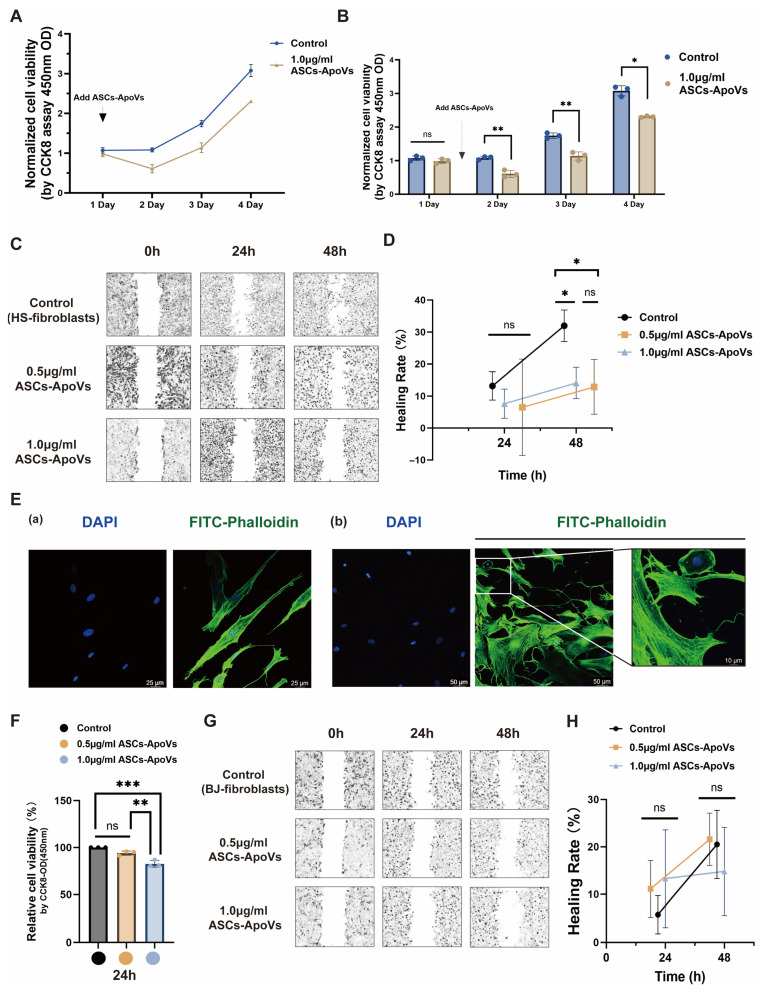
Evaluation of ASCs-ApoVs cytotoxicity at biologically active concentrations. (**A**,**B**) Effects of 1.0 µg/mL ASCs-ApoVs on the proliferation and overall viability of HS-fibroblasts assessed by one CCK-8 assay over a 3-day period (Mean ± SD, *n* = 3 independent experiments, data analyzed by Independent-samples *t*-test). (**C**) Images of scratch wound healing assay in HS-fibroblasts. (**D**) The quantified wound closure rates of HS-fibroblasts at 24 and 48 h (Mean ± SD, *n* = 3 independent experiments, data analyzed by one-way ANOVA with Tukey’s HSD). (**E**) Comparison of HS-fibroblasts’ cytoskeleton structure, stained with FITC (green)-phalloidin and DAPI (blue), without (**a**) and with (**b**) ASCs-ApoVs treatment (1.0 μg/mL, 24 h) (Scale bar = 10/25/50 μm). (**F**) CCK-8 assay (OD = 450 nm) measuring relative cell activity of BJ-fibroblasts following 24-h ASCs-ApoVs treatment. (Mean ± SD, *n* = 3 independent experiments, data analyzed by one-way ANOVA with Tukey’s HSD.) (**G**) The scratch assay images of normal skin-derived fibroblasts (BJ fibroblasts). (**H**) Wound healing rate quantification of (**G**) section. (Mean ± SD, *n* = 3 independent experiments, data analyzed by one-way ANOVA with Tukey’s HSD.) Statistical significance: ns *p* > 0.05, * *p* < 0.05, ** *p* < 0.01, *** *p* < 0.001.

In HS-fibroblasts, treatment with 1.0 μg/mL ASCs-ApoVs induced a marked and sustained reduction in both overall cell activity and proliferation, which was evident within the first 24 h and persisted for up to three days (*p* < 0.05 at least; Figure 2A,B). However, short-term (24 h) treatment with ASCs-ApoVs did not significantly affect cell migration ability (Figure 2C,D). A marked suppression of lateral migration was only observed after 48 h ASCs-ApoVs treatment at both concentration (*p* < 0.05; Figure 2C,D). Interestingly, FITC-phalloidin staining revealed that even the 24 h treatment had already induced distinct morphological changes in HS-fibroblasts, shifting their characteristic spindle shape (Figure 2E(a)) to an expanded and flattened phenotype (Figure 2E(b)), which likely impaired migration by enhancing cell adhesion. All these findings indicated that the inhibitory effect of ASCs-ApoVs on HS-fibroblast lateral migration is delayed relative to its effects on cytoskeleton morphology and cell proliferation.

In normal skin fibroblasts (BJ-fibroblasts), a concentration-dependent short-term (24 h) decrease in cell viability was also observed (*p* < 0.01; Figure 2F). However, their migratory capacity remained unaffected under all experimental conditions (Figure 2G,H). Disease-associated fibroblasts are known to exhibit distinct phenotypes and functions (possibly including vesicles uptake capacity), compared to normal fibroblasts [31,32]. We hypothesize that these intrinsic differences could offer a possible explanation for the observations and become a basis for developing targeted delivery systems in future ASCs-ApoVs administration.

In conclusion, as fibroblast proliferation and migration are critically involved in the pathogenesis of fibrosis [33], the ASCs-ApoVs induced alterations in HS-fibroblasts’ viability, proliferation and adhesion further contribute to the attenuation of hypertrophic scar.

### 3.3. Transcriptomic Profiling Identified CDC20 Downregulation as the Possible Central Target in ASCs-ApoVs Mediated HS-Fibroblasts Regulation

To further elucidate the underlying mechanisms by which ASCs-ApoVs exerts anti-scar effects, we performed transcriptome sequencing analysis, comparing HS-fibroblasts with ASCs-ApoVs (1.0 μg/mL, 24 h) treated ones.

**Figure 3:**
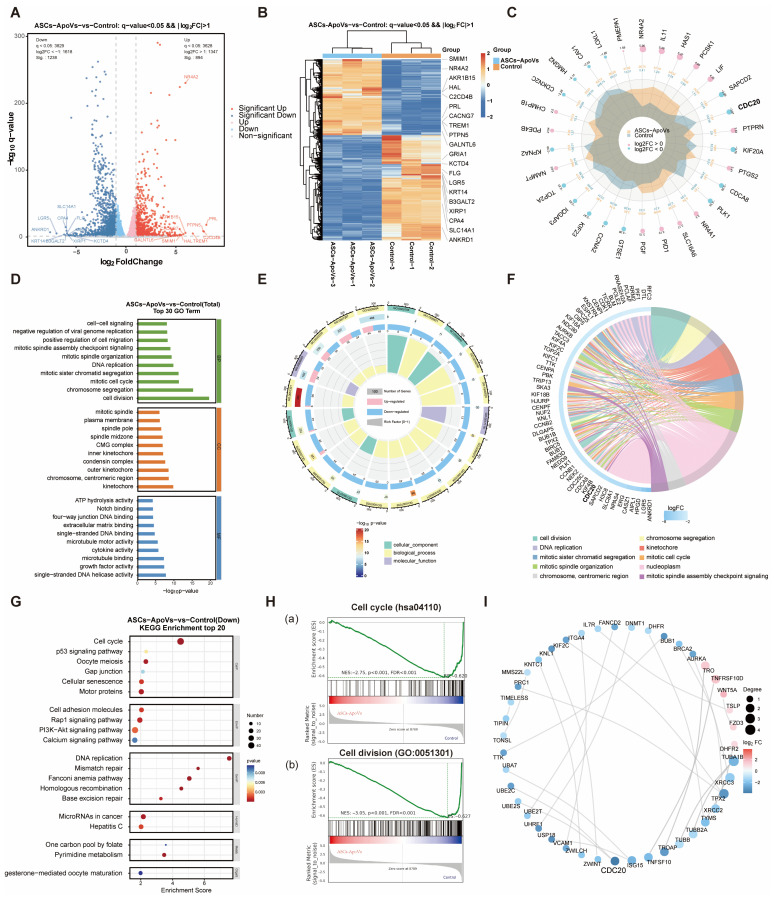
Transcriptional landscape of HS-fibroblasts in response to ASCs-ApoVs administration. (**A**) Volcano plot analysis of differentially expressed genes (DEGs) with or without ASCs-ApoVs treatment (1.0 μg/mL, 24 h). (**B**) Clustered heatmap of DEGs. (**C**) Radar chart visualization of TOP30 DEGs. (**D**) Bar chart visualization of TOP30 GO biological process enrichment. (**E**) Circular plot of GO enrichment of ASCs-ApoVs group vs. Control group. (**F**) Chord diagram of Top10 differentially down-regulated genes in each GO enrichment categories. (**G**) Bubble plot of Top20 enriched down-regulated KEGG pathways of HS-fibroblasts after ASCs-ApoVs treatment. (**H**) Gene Set Enrichment Analysis (GSEA) plots of (**a**) cell cycle and (**b**) cell division pathway after ASCs-ApoVs treatment. (**I**) The Protein-Protein Interaction (PPI) network analysis of Top30 differentially expressed protein-coding genes.

RNA-seq analysis demonstrated robust and reproducible transcriptional changes in HS-fibroblasts following ASCs-ApoVs treatment. Using the criteria of FDR < 0.05 and |log2FC| > 1, we identified a total of 2132 significantly DEGs (894 up-regulated and 1238 down-regulated. Volcano plot (Figure 3A) and enrichment heatmap (Figure 3B) revealed significant gene expression alterations following ASCs-ApoVs treatment. GO functional annotation identified cell cycle regulation, DNA replication, cell division, and cellular cytoskeleton proteins regulation as the most affected processes (Figure 3D). Among these, most biological processes were in down-regulation by GO enrichment visualization (Figure 3E). KEGG analysis further highlighted the suppression of cell cycle progression, DNA replication, and cytoskeletal proteins regulation (Figure 3G). GSEA also confirmed marked downregulation of cell cycle and cell division pathways (Figure 3H). Among all differentially expressed genes (DEGs), *CDC20* demonstrated both significant enrichment in KEGG and GO analyses (Figure 3F) and exhibited the most substantial fold change with highest statistical significance (Figure 3C). Besides, protein-protein interaction (PPI) network analysis of the top 30 differentially expressed protein-coding genes (Figure 3I) also highlighted CDC20 as a node, demonstrating robust interactions with other protein-encoding DEGs.

Together, ASCs-ApoVs treatment induced profound changes in the critical cellular processes of HS-fibroblasts, with the significant decrease in *CDC20* as a potential regulatory target.

### 3.4. CDC20 Down-Regulation Mediated the Conversion of HS-Fibroblasts Phenotypes

**Figure 4:**
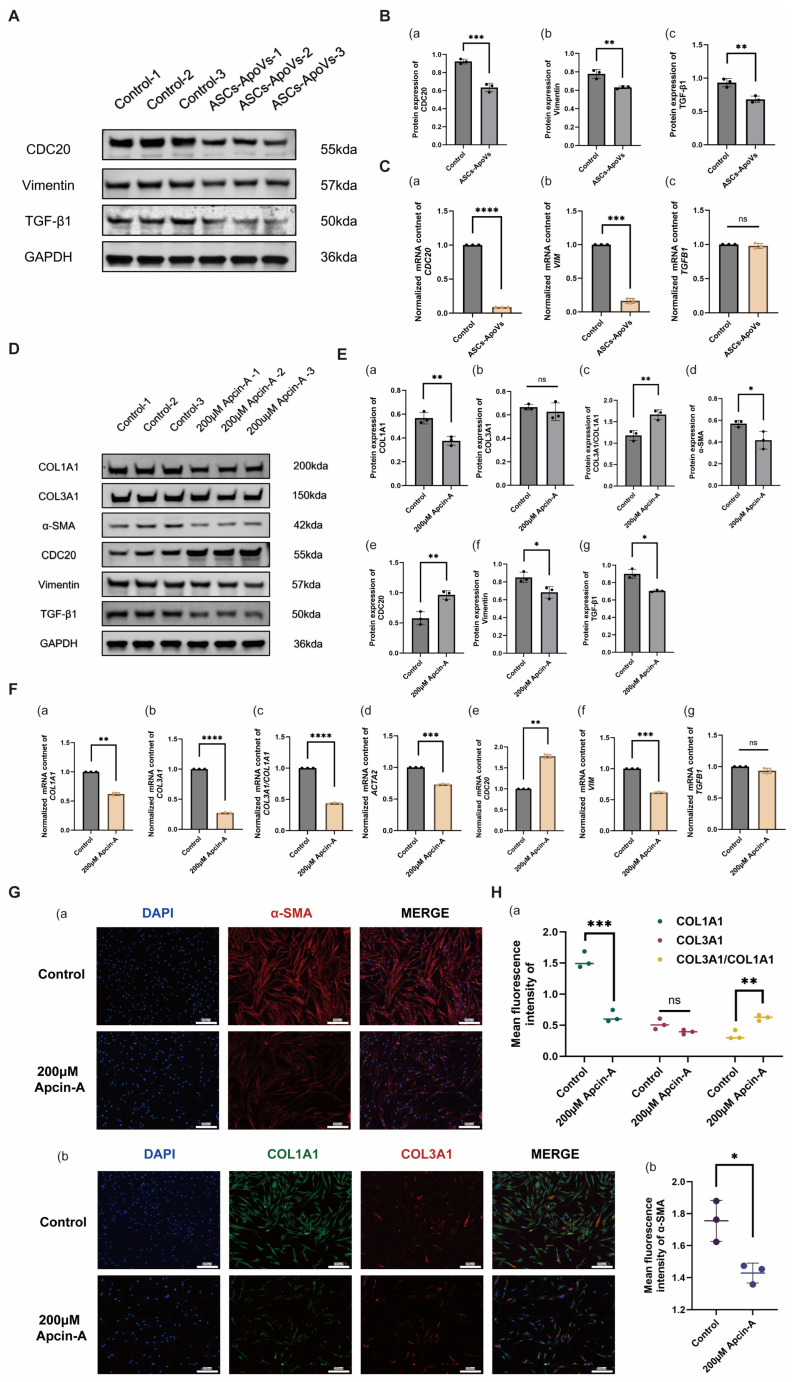
ASCs-ApoVs downregulated CDC20 as a pivotal regulatory node for hypertrophic scar regulation. (**A**) Western blot analysis of CDC20, Vimentin, and TGF-β1 expression in HS-fibroblasts after ASCs-ApoVs treatment (1.0 μg/mL, 24 h). (**B**) Quantification of (**a**) CDC20, (**b**) Vimentin, and (**c**) TGF-β1 expression from (**A**) by relative gray vale to GAPDH. (Mean ± SD, *n* = 3 independent experiments, data analyzed by Independent-samples *t*-test.) (**C**) Quantitative analysis of (**a**) *CDC20*, (**b**) *VIM*, and (**c**) *TGFB1* transcription by RT-qPCR, normalized to *GAPDH* and the control group. (Mean ± SD, *n* = 3 independent experiments, data analyzed by Independent-samples *t*-test.) (**D**) Western blot analysis of myofibroblast-related markers and CDC20 in HS-fibroblasts after Apcin-A administration (200 μM, 24 h). (**E**) Quantification of (**a**) COL1A1, (**b**) COL3A1, (**c**) COL3A1/COL1A1, (**d**) α-SMA, (**e**) CDC20, (**f**) Vimentin, and (**g**) TGF-β1 expression from (**D**) section by relative gray vale to GAPDH. (Mean ± SD, *n* = 3 independent experiments; data analyzed by Independent-samples *t*-test.) (**F**) Effect of Apcin-A (200 µM, 24 h) on the transcription of (**a**) *COL1A1*, (**b**) *COL3A1*, (**c**) *COL3A1/COL1A1*, (**d**) *ACTA2*, (**e**) *CDC20*, (**f**) *VIM*, and (**g**) *TGFB1* in HS-fibroblasts, measured by RT-qPCR. Quantitative analysis was normalized to *GAPDH* and the control group. (Mean ± SD, *n* = 3 independent experiments, data analyzed by Independent-samples *t*-test.) (**G**) Immunofluorescence staining of (**a**) α-SMA (red), (**b**) COL1A1 (green) and COL3A1 (red) in HS-fibroblasts with or without Apcin-A treatment (200 μM, 24 h) (Scale bar = 200 μm). (**H**) Scatter plots of mean fluorescence intensity of (**a**) COL1A1, COL3A1, and COL3A1/COL1A1, and (**b**) α-SMA, normalized to DAPI intensity. (Mean ± SD, *n* = 3 independent experiments, data analyzed by Independent-samples *t*-test.) Statistical significance: ns *p* > 0.05, * *p* < 0.05, ** *p* < 0.01, *** *p* < 0.001, **** *p* < 0.0001.

The down-regulation of CDC20 (*p* < 0.001 in Western blot; *p* < 0.0001 in RT-qPCR), Vimentin (*p* < 0.01 in Western blot and *p* < 0.001 in RT-qPCR) and TGF-β1 (*p* < 0.01 in Western blot; *p* > 0.05 in RT-qPCR) by ASCs-ApoVs, previously identified in the raw RNA-seq data, were verified by Western blot (Figure 4A,B) and RT-qPCR (Figure 4C).

To further confirm the role of CDC20 in the HS-fibroblasts regulation, we employed Apcin-A (a selective APC inhibitor that targets CDC20 to block substrate ubiquitination [34]) to partially mimic ASCs-ApoVs mediated CDC20 suppression. Western blot revealed significant suppression of COL1A1 (*p* < 0.01), α-SMA (*p* < 0.05), Vimentin (*p* < 0.05), and TGF-β1 (*p* < 0.05), with unchanged COL3A1 levels and increased COL3A1/COL1A1 value (*p* < 0.01) (Figure 4D,E). Immunofluorescence also indicated highly similar results (Figure 4G,H). RT-qPCR analysis further reconfirmed significant downregulation of *COL1A1* (*p* < 0.01), *ACTA2* (*p* < 0.001), and *VIM* (*p* < 0.001) at the transcriptional level (Figure 4F). However, a slight difference in *CDC20* and *COL3A1* transcription was observed in Apcin-A group compared with ASCs-ApoVs treatment.

Interestingly, both ASCs-ApoVs and Apcin-A significantly reduced TGF-β1 protein levels but did not alter its mRNA expression, suggesting a post-transcriptional regulatory mechanism. We hypothesize that both interventions modulate TGF-β1 via affecting its translational efficiency or protein stability. The detailed molecular mechanism remains to be investigated.

Beyond these phenotypes transformation, CDC20 inhibition could also directly attenuate HS-fibroblasts proliferation by disrupting its intrinsic cell cycle [35], mainly through G2/M phase arrest [36] (Figure S1F). The G2/M phase arrest, although less pronounced than CDC20 inhibitor treatment (200 µM Apcin-A, 24 h; Figure S1F(c)), was also observed following ASCs-ApoVs administration (1.0 µg/mL, 24 h; Figure S1F(b)). Because ASCs-ApoVs could only significantly inhibit CDC20 synthesis, their functional impact on already accumulated CDC20 may be less pronounced compared to that of Apcin-A. Therefore, we hypothesized that the attenuated efficacy of G2/M phase arrest by ASCs-ApoVs was likely due to the presence of functional CDC20 that had accumulated prior to ApoVs intervention [37].

In summary, CDC20 downregulation has been established as a pivotal node through which ASCs-ApoVs regulate HS-fibroblasts, functioning by not only directly disrupting cell cycle progression but also driving phenotypic conversion.

### 3.5. ASCs-ApoVs Mediated CDC20 Inhibition Attenuated Wnt/β-Catenin Signaling in HS-Fibroblasts

The Wnt/β-catenin signaling pathway is a well-established therapeutic target for hypertrophic scar modulation [38,39]. Given that CDC20 inhibition induced Wnt/β-catenin pathway suppression has been documented in renal fibrosis [40] and cutaneous squamous cell carcinoma (cSCC) [41], we tested whether Wnt/β-catenin signaling is also blocked in ASCs-ApoVs treated HS-fibroblasts.

**Figure 5:**
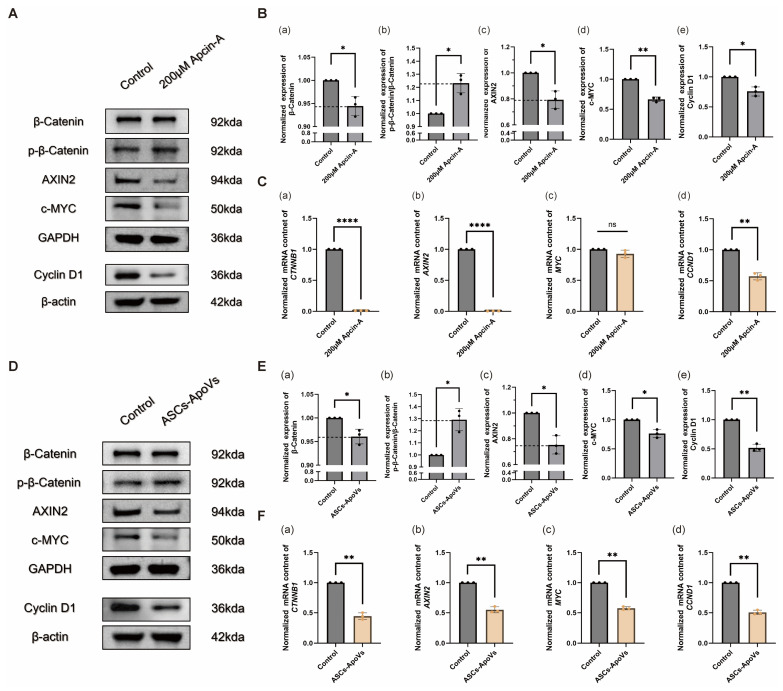
CDC20 inhibition attenuated myofibroblasts’ phenotypes via Wnt/β-catenin signaling suppression. (**A**) Western blot of Wnt/β-catenin signaling pathway related indexes in HS-fibroblasts after Apcin-A treatment (200 μM, 24 h). (**B**) Quantification of (**a**) β-Catenin, (**b**) p-β-Catenin, (**c**) AXIN2, (**d**) c-MYC, and (**e**) Cyclin D1 expression from (**A**) section by relative gray vale to GAPDH for most indexes and β-actin for Cyclin D1. (Mean ± SD, *n* = 3 independent experiments, data analyzed by Independent-samples *t*-test.) (**C**) Quantitative analysis of (**a**) *CTNNB1*, (**b**) *AXIN2*, (**c**) *MYC*, (**d**) *CCND1* transcription by RT-qPCR, normalized to *GAPDH* and the control group. (Mean ± SD, *n* = 3 independent experiments, data analyzed by Independent-samples *t*-test.) (**D**) Western blot of Wnt/β-catenin signaling pathway related indexes in HS-fibroblasts after ASCs-ApoVs treatment (1.0 μg/mL, 24 h). (**E**) Quantification of (**a**) β-Catenin, (**b**) p-β-Catenin, (**c**) AXIN2, (**d**) c-MYC, and (**e**) Cyclin D1 expression from (**D**) section by relative gray vale to GAPDH for most indexes and β-actin for Cyclin D1. (Mean ± SD, *n* = 3 independent experiments, data analyzed by Independent-samples *t*-test.) (**F**) Quantitative analysis of (**a**) *CTNNB1*, (**b**) *AXIN2*, (**c**) *MYC*, (**d**) *CCND1* transcription by RT-qPCR, normalized to *GAPDH* and the control group. (Mean ± SD, *n* = 3 independent experiments, data analyzed by Independent-samples *t*-test.) Statistical significance: ns *p* > 0.05, * *p* < 0.05, ** *p* < 0.01, **** *p* < 0.0001.

Downregulation of Wnt/β-catenin pathway markers was confirmed in HS-fibroblasts treated with Apcin-A (200 μM, 24 h). Specifically, the reduction in β-catenin protein and mRNA levels (*p* < 0.05 for Western blot; *p* < 0.0001 for RT-qPCR), along with an increased ratio of phosphorylated β-catenin (*p* < 0.05, Western blot), indicated suppression of the Wnt/β-catenin signaling pathway. Furthermore, decreased expression of AXIN2 [42] (*p* < 0.05 in Western blot, *p* < 0.0001 in RT-qPCR), c-MYC [43] (*p* < 0.01 in Western blot; *p* > 0.05 in RT-qPCR), and Cyclin D1 [44] (*p* < 0.05 in Western blot, *p* < 0.01 in RT-qPCR) was also observed (Figure 5A–C).

Consistent with Apcin-A, ASCs-ApoVs (1.0 μg/mL, 24 h) also significantly suppressed the protein and mRNA content of key Wnt/β-catenin pathway effectors, accompanied with a marked increase in phosphorylated β-catenin (*p* < 0.05 in Western blot) and consequently enhanced β-catenin degradation (Figure 5D–F).

In summary, our findings confirmed the pivotal role of CDC20-downregulation and therefore Wnt/β-catenin signaling pathway inhibition as the mechanism of ASCs-ApoVs against HS-fibroblasts.

### 3.6. Characteristics of ASCs-ApoVs Under Delivery Conditions

For clinical translation, STS-induced ASCs-ApoVs demonstrated favorable stability and potent bioactivity, underscoring therapeutic advantages that distinguish them from other extracellular vesicle subtypes.

**Figure 6:**
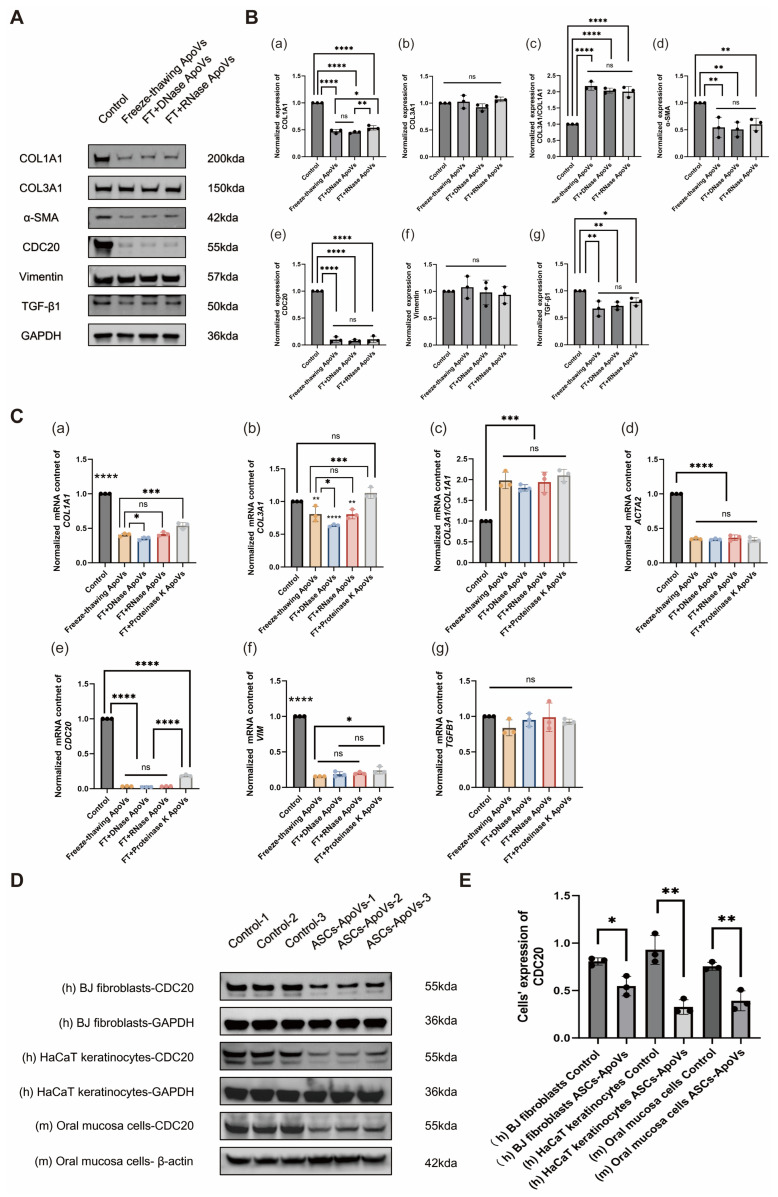
ASCs-ApoVs exhibited cross-species conservation and robust environmental resilience. (**A**) Western blot analysis of myofibroblast-related markers in HS-fibroblasts with freezing-thawing (for 3 times) ASCs-ApoVs, freezing-thawing ASCs-ApoVs with additional DNase I treatment, freezing-thawing ASCs-ApoVs with additional RNase A treatment. (**B**) Quantification of (**a**) COL1A1, (**b**) COL3A1, (**c**) COL3A1/COL1A1, (**d**) α-SMA, (**e**) CDC20, (**f**) Vimentin, and (**g**) TGF-β1 expression from (**A**) section by relative gray vale to GAPDH and the control group. (Mean ± SD, *n* = 3 independent experiments, data analyzed by one-way ANOVA with Tukey’s HSD.) (**C**) RT-qPCR results of (**a**) *COL1A1*, (**b**) *COL3A1*, (**c**) *COL3A1/COL1A1*, (**d**) *ACTA2*, (**e**) *CDC20*, (**f**) *VIM*, and (**g**) *TGFB1* in HS-fibroblasts with processed ASCs-ApoVs treatment (ASCs-ApoVs freezing-thawing for 3 times, freezing-thawing ASCs-ApoVs with additional DNase I treatment, freezing-thawing ASCs-ApoVs with additional RNase A treatment, freezing-thawing ASCs-ApoVs with additional Proteinase K treatment), normalized to *GAPDH* and the control group. (Mean ± SD, *n* = 3 independent experiments, data analyzed by one-way ANOVA with Tukey’s HSD.) (**D**) Western blot analysis of CDC20 expression in normal human fibroblasts (BJ-fibroblasts), human keratinocytes (HaCaT cells), and mouse oral mucosa epithelial cells. (**E**) Quantification of CDC20 expression from (**D**) section by relative gray vale to GAPDH/β-actin. (Mean ± SD, *n* = 3 independent experiments, data analyzed by Independent-samples *t*-test.) Statistical significance: ns *p* > 0.05, * *p* < 0.05, ** *p* < 0.01, *** *p* < 0.001, **** *p* < 0.0001.

In Western blot analysis, ASCs-ApoVs retained potent HS-fibroblasts modulating activity despite several freeze-thaw cycles and different enzymatic treatment (DNase I and RNase A), maintaining strong suppression of COL1A1 (*p* < 0.0001), α-SMA (*p* < 0.01 at least), CDC20 (*p* < 0.0001) and TGF-β1 (*p* < 0.05 at least) while preserving the beneficial COL3A1/COL1A1 value (*p* < 0.0001) (Figure 6A,B). RT-qPCR analysis re-confirmed ASCs-ApoVs’ exceptional stability against freeze-thaw cycles and enzymatic challenges, maintaining strong suppression of *COL1A1* (*p* < 0.0001), *ACTA2* (*p* < 0.0001), *CDC20* (*p* < 0.0001) and *VIM* (*p* < 0.0001) transcription while preserving the beneficial *COL3A1/COL1A1* value (*p* < 0.001) (Figure 6C). However, proteinase K significantly attenuated the inhibitory effect of ASCs-ApoVs on *COL1A1* (*p* < 0.001), *CDC20* (*p* < 0.0001), and *VIM* (*p* < 0.05) transcription. Besides, ASCs-ApoVs with DNase I treatment significantly inhibited the transcription of *COL3A1* (*p* < 0.0001). The observed response to enzyme treatments implies the possible presence of active and surface-localized components on ASCs-ApoVs.

Figure 2 showed ASCs-ApoVs’ impact on both activated and quiescent fibroblasts subtypes, suggesting its broad-spectrum activity beyond targeted intervention. This feature was further evidenced by consistent CDC20 downregulation across multiple cell types, including human BJ fibroblasts (*p* < 0.05), human HaCaT keratinocytes (*p* < 0.01), and mouse oral mucosa epithelium cells (*p* < 0.01) following ASCs-ApoVs treatment (1.0 μg/mL, 24 h) (Figure 6D,E). We propose that the generic uptake ability of ASCs-ApoVs and evolutionarily conservative mechanism leads to the consistent disruption of CDC20 expression in different recipient cells.

In summary, the favorable stability of ASCs-ApoVs supports their clinical translation. And the non-specificity, while indicating its broad potential for treating fibrotic and CDC20/Wnt/β-catenin related diseases, also presents a challenge for precision medicine.

### 3.7. In Vivo Therapeutic Efficacy in a Rabbit Ear Hypertrophic Scar Model

To further evaluate the in vivo scar-modulating capabilities of ASCs-ApoVs, rabbit ear hypertrophic scar model has been conducted.

**Figure 7:**
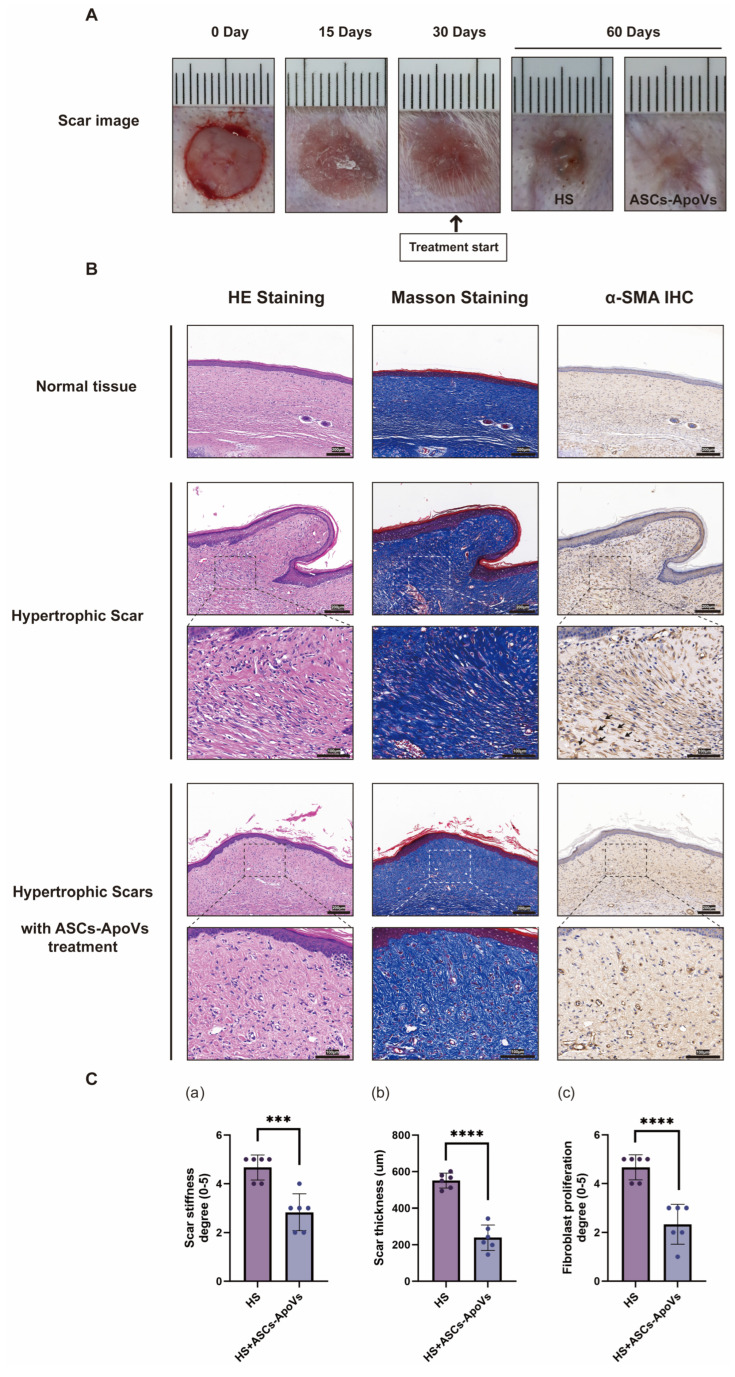
Histological staining of hypertrophic scars with ASCs-ApoVs treatment. (**A**) Photographs of the rabbit ear hypertrophic scar model. (**B**) Histological results of H&E, Masson’s Trichrome, and immunohistochemical staining of α-SMA in normal skin tissues, hypertrophic scar group (with PBS injection at same volume as control), and ASCs-ApoVs (1.0 μg/mL, 24 h) treated group (Scale bar = 100/200 μm). (**C**) Analysis of (**a**) stiffness degree of hypertrophic scars, (**b**) thickness of hypertrophic scars, and (**c**) proliferation degree of fibroblasts. (Mean ± SD, *n* = 6 independent scar experiments, statistical significance was determined by Independent-samples *t*-test.) Statistical significance: *** *p* < 0.001, **** *p* < 0.0001.

The induced scars exhibited typical hypertrophic characteristics: raised and stiff tissue protruding from the surrounding normal skin. Histological assessment of H&E-stained sections revealed hypercellularity and locally disorganized, whorl-like fibrous structures. α-SMA immunohistochemistry showed intense staining within proliferated fibroblasts, which displayed irregular morphology consistent with activated myofibroblasts (as arrow shows) (Figure 7B-Hypertrophic scar).

Local injection of ASCs-ApoVs (30 μL per scar, 1.0 μg/mL) significantly reduced scar stiffness (*p* < 0.001) and thickness (*p* < 0.0001) (Figure 7C). Although the ASCs-ApoVs treatment group still partially exhibited morphological and textural differences compared to the surrounding tissue, the tissue appeared smoother and exhibited less pronounced redness and swelling than the hypertrophic scar control group treated with PBS (Figure 7A). Tissue sections demonstrated markedly decreased cellularity (*p* < 0.0001) and α-SMA positive fibroblasts. However, ASCs-ApoVs did not significantly reduce collagen staining as assessed by Masson’s trichrome, but they did play a role in regulating collagen structure and arrangement, leading to a substantial reduction in whorl-like fibrous arrangements. Given that ASCs-ApoVs strongly suppressed α-SMA expression, this observation suggests that ASCs-ApoVs may primarily inhibit fibrotic activity and modulate collagen allocation rather than degrade pre-existing collagen. Meanwhile, no abnormalities have been observed in rabbits’ tissues or organ functions, confirming the biosafety of ASCs-ApoVs at this physiological concentration.

In summary, the anti-fibrotic effect of ASCs-ApoVs was confirmed in an animal model, demonstrating its potential as a promising therapeutic strategy for hypertrophic scar treatment.

## 4. Discussion

Traditionally, extracellular vesicles (EVs) are categorized into apoptotic bodies (AB/ApoBDs; 1000 to 5000 nm) [29], microvesicles (or microparticles), and exosomes [45]. Recently, apoptotic extracellular vesicles (ApoEVs) or apoptotic microvesicles (ApoMVs) [46] in (200–1000 nm) as well as apoptotic exosomes (ApoExos) in (30–100 nm) [47,48] have also been found. In this study, the vesicles’ size distribution predominantly corresponds to ApoMVs [49] and definitely without tinier ApoExos [48], but unable to fully rule out the presence of physiologically released microvesicles. Therefore, we adopted the term “apoptotic vesicles (ApoVs)” to describe our productions while distinguishing them from previously established categories.

Hypertrophic scar (HS) are characterized by excessive dermal myofibroblast proliferation and aberrant collagen deposition, resulting in raised scar tissue [50]. In cellular experiment (Figure 1 and Figure 4A,B), adipose stem cells derived apoptotic vesicles (ASCs-ApoVs) modulated both the ratio and absolute levels of COL1A1 and COL3A1, thereby promoting a softer scar phenotype [51]. It also suppressed α-SMA expression in HS-fibroblasts, attenuating myofibroblast-driven collagen contraction [52]. Furthermore, ASCs-ApoVs inhibited Vimentin expression, mitigating epithelial-mesenchymal transition (EMT)-mediated fibrosis [53]; and downregulated TGF-β1, one of central mediators in fibrotic diseases [54]. Animal experiments (Figure 7) have further demonstrated its significant regulatory effects on scar thickness, scar hardness, and α-SMA positive fibroblasts within hypertrophic scar tissue. Collectively, this study confirmed the efficacy of ASCs-ApoVs in anti-fibrosis, especially for treating hypertrophic scar.

Compared with other conventional approaches for HS regulation, ApoVs exhibits remarkable advantages. Mechanically speaking, ApoVs enables comprehensive therapeutic effects, demonstrating potential superiority than solely Apcin-A administration in several indexes. Structurally speaking, the nanoscale diameter of ApoVs (Figure S1E) facilitates enhanced biodistribution and has the ability to traverse biological barriers [55]—features usually unattainable with intact cellular therapies [56]. Besides, the phospholipid bilayer of ApoVs could provide cargo protection (Figure 6A–C), significantly improving molecular bioavailability [57]. Clinically, ApoVs exhibit higher yields [58], simplified production (e.g., concentrated induction of apoptosis by STS), and easier storage and logistics compared with other cell-free therapies—all of which endow them with economic viability and clinical translation potential.

ApoVs induced significant and stable phenotypic modulation of HS-fibroblasts is mainly attributed to the down-regulation of CDC20 (Figure 4D,E) and subsequent inhibition of the Wnt/β-catenin signaling pathway activity (Figure 5). Based on studies across renal [40], hepatic [59], cardiac [60], and pulmonary [61] fibrosis, CDC20 upregulation acts as a critical downstream node responsive to multiple pro-fibrotic stimuli (such as TGF-β1 and radiation) [62]. Elevated CDC20 could directly enhance anti-apoptotic signaling [60]; or activate β-catenin [40] to promote epithelial-mesenchymal transition (EMT) [59], potentially through the ubiquitination process [61]. Our results demonstrated that ASCs-ApoVs mediated CDC20 downregulation also curbed fibrotic progression via a dual mechanism: first, by directly inducing cell-cycle arrest to inhibit HS-fibroblasts proliferation (Figure 2A,B and Figure S1F); and second, by suppressing the Wnt/β-catenin pathway (Figure 5D–F), thereby promoting a phenotypic switch in HS-fibroblasts.

Wnt/β-catenin signaling is a well-established driver of hypertrophic scar progression [63,64]. In this study, we established a causal link between CDC20 inhibition and the suppression of Wnt/β-catenin pathway (Figure 5A–C) in the context of HS. While the precise mechanism requires further investigation, we postulate that the CDC20-APC/C complex [65] may attenuate Wnt/β-catenin signaling by targeting its positive/negative regulators or directly influencing β-catenin stability. Fully delineating this mechanism represents a critical next step, promising to reveal novel regulators of the Wnt/β-catenin pathway and to uncover critical links between cell cycle progression and typical fibrotic signaling.

While ApoVs hold considerable therapeutic promise, their clinical translation must address several challenges, including cost control, the establishment of Good Manufacturing Practice (GMP) standards to ensure consistency and safety, and improvements in targeting accuracy—challenges underscored by the cross-species inhibition of CDC20 (Figure 6D,E) and unintended upregulation of TGF-β1 in HaCaT cells (Figure S2A,B). Future strategies to facilitate the application of ApoVs in disease treatment may involve the biological engineering and the use of material-assisted controlled delivery systems. In addition, establishing consensus on stem cell in vitro culture protocols and standardizing the commercial production of extracellular vesicles will provide a clear and feasible pathway forward.

It is important to acknowledge several limitations in this study. First, the precise mechanisms through which ASCs-ApoVs mediate CDC20 downregulation in recipient cells remain incompletely elucidated. Identifying the bioactive components responsible for this effect in ASCs-ApoVs warrants further investigation. Second, the molecular link between CDC20 reduction and the observed inhibition of Wnt/β-catenin signaling remains unclear. Given the known fluctuations in Wnt/β-catenin pathway activity during the cell cycle [66], this area represents a promising direction for future research. Third, the post-transcriptional mechanism underlying TGF-β1 downregulation by ASCs-ApoVs and Apcin-A has not been elucidated. While the discordance between protein and mRNA levels points to translational or stability regulation, future studies are needed to dissect the precise pathway. Finally, the inherent non-selective function of ASCs-ApoVs could be further optimized through various strategies in future studies, and findings obtained from immortalized cell lines such as HaCaT require further validation in primary human cells.

## Figures and Tables

**Table 1:** Primer information.

Gene	Species	Forward	Reverse
*COL1A1*	Human	5′ AGG GCC AAG ACG AAG ACA TC 3′	5′ GTC GGT GGG TGA CTC TGA GC 3′
*COL3A1*	Human	5′ TGA AGG GCA GGG AAC AAC T 3′	5′ GGA TGA AGC AGA GCG AGA AG 3′
*ACTA2*	Human	5′ CGT GGC TAT TCC TTC GTT ACT A 3′	5′ ATC AGG CAA CTC GTA ACT CTT C 3′
*VIM*	Human	5′ CTG TAA GTT GGT AGC ACT GAG 3′	5′ TTA GGG GAA ACC GTT AGA C 3′
*TGFB1*	Human	5′ TAC TAC GCC AAG GAG GTC AC 3′	5′ GAG AGC AAC ACG GGT TCA G 3′
*CDC20*	Human	5′ ATT CCT TCC CTG CCA GAC 3′	5′ GCC AGT ACA TTC CCA GAA CTC 3′
*CTNNB1*	Human	5′ TCT CTG AGT GGT AAA GGC AAT C 3′	5′ TGA GCA GCA TCA AAC TGT GT 3′
*AXIN2*	Human	5′ GAA ATG CGT GGA TAC CTT AGA C 3′	5′ GGA ATC AAT CTG CTG CTT CTT 3′
*MYC*	Human	5′ CGG AAA CGA CGA GAA CAG T 3′	5′ CAT AGG TGA TTG CTC AGG ACA T 3′
*CCND1*	Human	5′ CCC CTT CCA TCT CTG ACT TA 3′	5′ CCT CTA TCA TCT GTA GCA CAA CC 3′
*GAPDH*	Human	5′ GGG AAG GTG AAG GTC GGA GT 3′	5′ GGG GTC ATT GAT GGC AAC A 3′

**Table 2:** Antibody information.

Antibodies	Catalog Number	Dilution Ratio
COL1A1 antibody	Proteintech 67288-1-Ig	1:5000
COL3A1 antibody	ABclonal A0817	1:5000
α-SMA antibody	Affinity AF1032	1:1000
Vimentin antibody	CST #P08670	1:10,000
TGF-β1 antibody	ABclonal A25313	1:1000
CDC20 antibody	Proteintech 10252-1-AP	1:3000
p-β-catenin antibody	Affinity DF2989	1:1000
β-catenin antibody	Proteintech 51067-2-AP	1:1000
AXIN2 antibody	Proteintech 20540-1-AP	1:1000
c-MYC antibody	Proteintech 67447-1-Ig	1:1000
Cyclin D1 antibody	Proteintech 60186-1-Ig	1:1000
GAPDH antibody	Proteintech 60004-1-Ig	1:10,000
β-actin antibody	Proteintech 66009-1-Ig	1:10,000
CD34 antibody	Proteintech 14486-1-AP	1:1000
CD105 antibody	Invitrogen MA5-17041	1:1000
HRP-conjugated Goat Anti-Rabbit IgG	Proteintech SA00001-2	1:10,000
HRP-conjugated Goat Anti-Mouse	Proteintech SA00001-1	1:10,000
CoraLite488-conjugated Goat Anti-Mouse IgG	Proteintech SA00013-1	1:1000
CoraLite594-conjugated Goat Anti-Rabbit IgG	Proteintech SA00013-4	1:1000
CoraLite488-conjugated Goat Anti-Rabbit IgG	Proteintech SA00013-2	1:1000
CoraLite594-conjugated Goat Anti-Mouse IgG	Proteintech SA00013-3	1:1000

## Data Availability

The raw transcriptome sequencing data are available in the NCBI database under accession number: PRJNA1348794. Other data supporting the findings of this study are available from the corresponding author upon reasonable request.

## Supplementary Materials

The following supporting information can be downloaded at: https://www.mdpi.com/article/10.3390/biomedicines14051083/s1, Figure S1. Characterization of adipose stem cells (ASCs) and ASCs derived apoptotic vesicles (ASCs-ApoVs). Figure S2. ASCs-ApoVs’ anti-fibrosis regulation was not recipient cell specific. References [67,68,69] are cited in the Supplementary Materials.
