# Supplementary material for: Adipose Stem Cell-Derived Apoptotic Vesicles Attenuate Hypertrophic Scarring by Targeting the CDC20/WNT Signaling Pathway
Source: Biomedicines. 2026 May 11;14(5):1083. doi: 10.3390/biomedicines14051083 (PMC13204812; doi:10.3390/biomedicines14051083)
Supplement: Supplementary file 1 [file biomedicines-14-01083-s001.zip › biomedicines-4248945-supplementary.pdf]

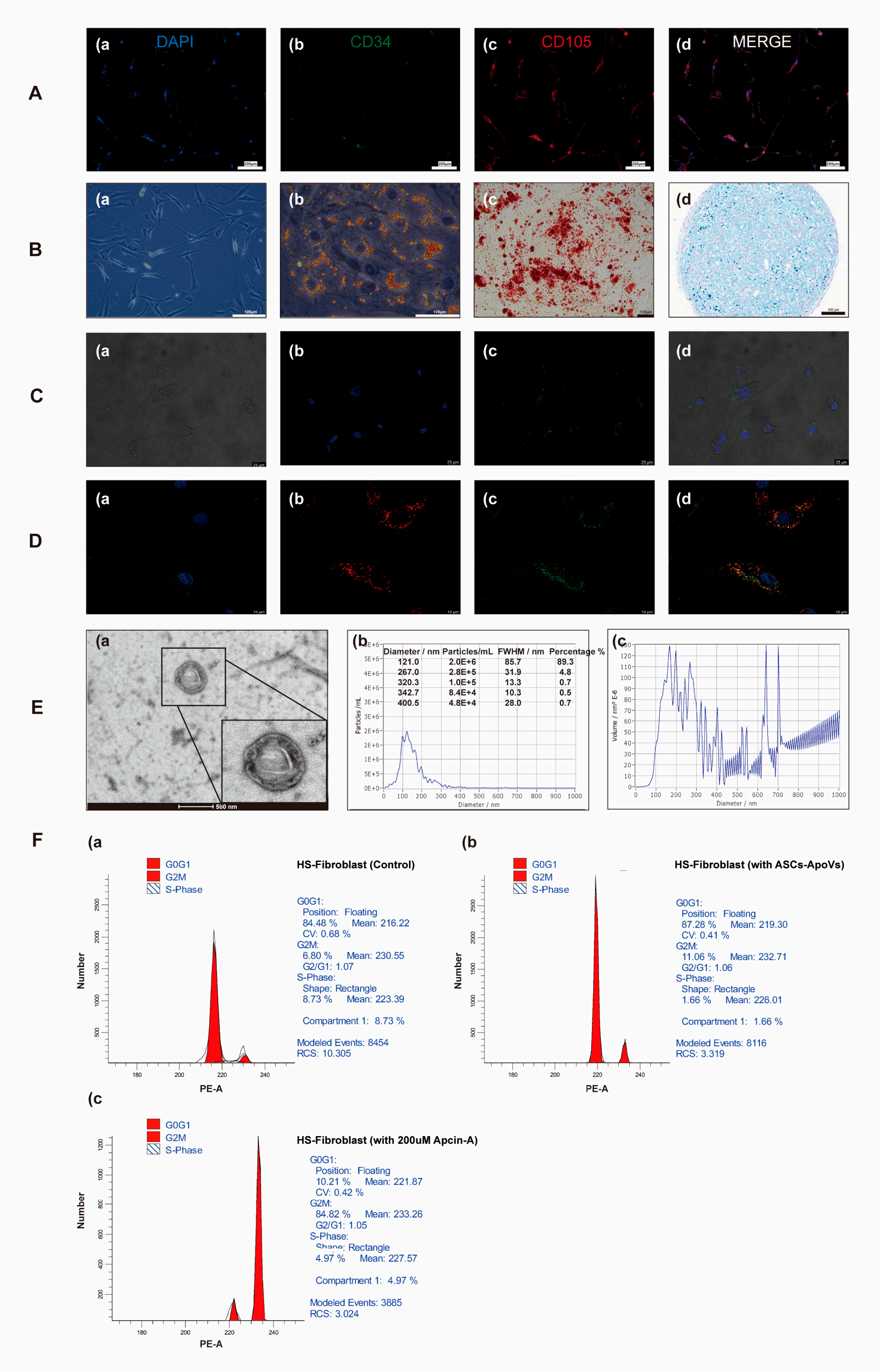
A
B
C
D
E
F
(a
DAPI
(b
CD34
(c
CD105
(d
MERGE
500 nm
Diameter / nm Particles/mL FWHM / nm Percentage %
121.0 2.0E+6 85.7 89.3
267.0 2.8E+5 31.9 4.8
320.3 1.0E+5 13.3 0.7
342.7 8.4E+4 10.3 0.5
400.5 4.8E+4 28.0 0.7
Particles /mL
Diameter / nm
Volume / nm³ E-6
G0G1
G2M
S-Phase
HS-Fibroblast (Control)
G0G1:
Position: Floating
84.48 % Mean: 216.22
CV: 0.68 %
G2M:
6.80 % Mean: 230.55
G2/G1: 1.07
S-Phase:
Shape: Rectangle
8.73 % Mean: 223.39
Compartment 1: 8.73 %
Modeled Events: 8454
RCS: 10.305
Number
PE-A
HS-Fibroblast (with ASCs-ApoVs)
G0G1:
Position: Floating
87.28 % Mean: 219.30
CV: 0.41 %
G2M:
11.06 % Mean: 232.71
G2/G1: 1.06
S-Phase:
Shape: Rectangle
1.66 % Mean: 226.01
Compartment 1: 1.66 %
Modeled Events: 8116
RCS: 3.319
HS-Fibroblast (with 200uM Apcin-A)
G0G1:
Position: Floating
10.21 % Mean: 221.87
CV: 0.42 %
G2M:
84.82 % Mean: 233.26
G2/G1: 1.05
S-Phase:
Shape: Rectangle
4.97 % Mean: 227.57
Compartment 1: 4.97 %
Modeled Events: 3885
RCS: 3.024

Figure S1. Characterization of adipose stem cells (ASCs) and ASCs derived apoptotic vesicles (ASCs-ApoVs). (A) Immunofluorescence characterization of adipose stem cells (ASCs) cultured on coverslips, showing negative CD34 [67], positive CD105 [68], and nuclei (blue, DAPI) staining (Scale bar = 200 μm). **(B)** Multilineage differentiation potential test of ASCs. **(a** Phase-contrast morphology of undifferentiated ASCs. **(b** Oil Red staining of ASCs following 2-week adipogenic differentiation (lipid droplets in red are visible in ASCs). **(c** Alizarin Red S Staining of ASCs after 3-week osteogenic differentiation (mineralized nodules in red are visible in filed). **(d** Alcian Blue Staining with Nuclear Fast Red after 5-week chondrogenic differentiation of ASCs (representative paraffin section of soft cartilage ball with glycosaminoglycans in blue and nuclear in red). (Scale bar = 100 μm). **(C)** Internalization of ASCs-ApoVs by HS-fibroblasts. **(a** Phase-contrast morphology of HS-fibroblasts treated with ASCs-ApoVs (1.0 μg/ml, 2h). **(b** Nuclear staining (Hoechst, blue) of the HS-fibroblasts. **(c** ASCs-ApoVs was labeled with DiO dyestuff (green). **(d** Merged image of (a-(c, demonstrating ASCs-ApoVs were taken in by HS-fibroblasts (Scale bar = 25 μm). **(D)** Subcellular localization of internalized ASCs-ApoVs in HS-fibroblasts. **(a** Nuclear staining (Hoechst, blue) of ASCs-ApoVs treated HS-fibroblasts. **(b** Membrane staining (CM-Dil, red) of cellular organelles of HS-fibroblasts. **(c** ASCs-ApoVs were labeled with DiO dyestuff (green). **(d** Merged image showing dual localization patterns: some ASCs-ApoVs co-localized with membranous organelles (yellow in merged channels); others dispersed in cytoplasm (green only) (Scale bar = 10 μm). **(E)** Apoptotic vesicles (ApoVs) from ASCs. **(a** Transmission electron microscopy (TEM) results of ASCs-ApoVs, revealing classic vesicular structures with visible outer membrane structures consistent with a phospholipid bilayer [69] (Scale bar = 500 μm). **(b-c** Nanoparticle tracking analysis (NTA) showed the particle diameter of ASCs-ApoVs. **(F)** Cell cycle distribution analysis with propidium iodide (PI) staining by flow cytometry. **(a** HS-fibroblasts with HD-DMEM (control group). **(b** HS-fibroblasts with ASCs-ApoVs (1.0 μg/ml, 24h). **(c** HS-fibroblasts with Apcin-A (200 μM, 24h).

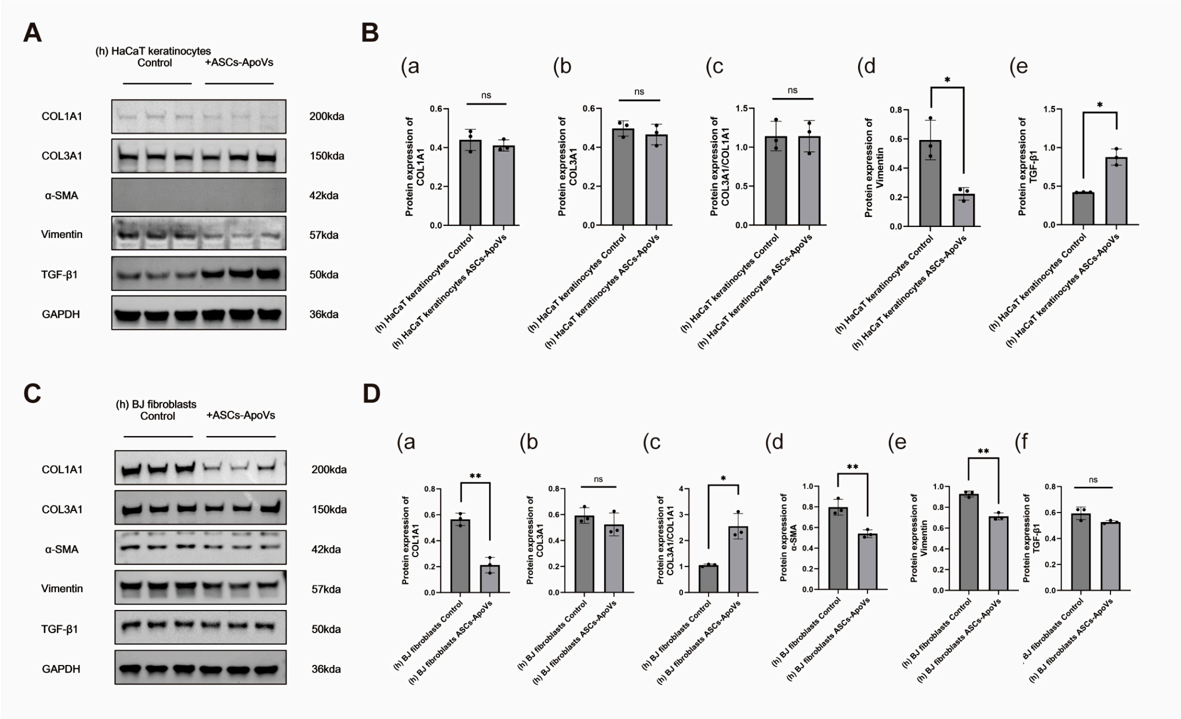

Figure S2. ASCs-ApoVs' anti-fibrosis regulation was not recipient cell specific. (A) Western blot analysis of myofibroblast-related markers in human HaCaT keratinocytes after ASCs-ApoVs treatment (1.0 μg/ml, 24h). **(B)** Quantification of protein expression from (A) section by relative gray vale to GAPDH. (Mean ± SD, n=3 independent experiments, data analyzed by Independent-samples t-test.) **(C)** Western blot analysis of myofibroblast-related markers in normal human fibroblasts (BJ-fibroblasts) after ASCs-ApoVs treatment (1.0 μg/ml, 24h). **(D)** Quantification of protein expression from (C) section by relative gray vale to GAPDH. (Mean ± SD, n=3 independent experiments, data analyzed by Independent-samples t-test.) Statistical significance: *$p < 0.05$, **$p < 0.01$, ***$p < 0.001$, ****$p < 0.0001$.

Reference

67. Madhu, V.; Kilanski, A.; Reghu, N.; Dighe, A.S.; Cui, Q. Expression of CD105 and CD34 Receptors Controls BMP-induced in Vitro Mineralization of Mouse Adipose-derived Stem Cells but Does Not Predict Their in Vivo Bone-forming Potential. *J. Orthop. Res.* **2015**, *33*, 625–632. https://doi.org/10.1002/jor.22883.

68. Kazemi, F.; Sadeghian, F.; Pirsadeghi, A.; Asadi, F.; Javdani, H.; Yousefi-Ahmadipour, A. Adipose Mesenchymal Stem Cell Conditioned Medium and Extract: A Promising Therapeutic Option for Regenerative Breast Cancer Therapy. *SAGE Open Med.* **2024**, *12*, 20503121241306606. https://doi.org/10.1177/20503121241306606.

69. Gregory, C.D.; Rimmer, M.P. Extracellular Vesicles Arising from Apoptosis: Forms, Functions, and Applications. *J. Pathol.* **2023**, *260*, 592–608. https://doi.org/10.1002/path.6138.
